# *Toxoplasma gondii* infections among pregnant women, children and HIV-seropositive persons in Accra, Ghana

**DOI:** 10.1186/s41182-016-0018-5

**Published:** 2016-06-01

**Authors:** Irene Ayi, Augustine Odoi-Kpoti Sowah, Emmanuel Awusah Blay, Takashi Suzuki, Nobuo Ohta, Patrick F. Ayeh-Kumi

**Affiliations:** Department of Parasitology, Noguchi Memorial Institute for Medical Research, College of Health Science, University of Ghana, P. O. Box LG 581, Legon, Accra, Ghana; Korle-Bu Central Laboratory, Korle-Bu Teaching Hospital, P. O. Box 77, Korle-Bu, Accra, Ghana; Department of Environmental Parasitology, Graduate School of Medical and Dental Sciences, Tokyo Medical and Dental University, 1-5-45 Yushima, Bunkyo-ku, Tokyo, 113-8519 Japan; School of Biological and Allied Health Sciences, College of Health Science, University of Ghana, Korle-Bu, Accra, Ghana

**Keywords:** *Toxoplasma gondii*, Anti-*Toxoplasma gondii* antibodies, Immune-compromised

## Abstract

**Background:**

*Toxoplasma gondii* infection can lead to severe disease outcomes in immune-compromised people. This study sought to determine the seroprevalence of anti-*T. gondii* antibodies among pregnant women, hospitalized children (<5 years old) and HIV-seropositive persons in Accra.

**Methods:**

A cross-sectional study was conducted in two hospitals in Accra, and a total of 450 voluntary participants were recruited for the study consisting of 125 pregnant women, 200 children and 125 HIV-seropositive persons. Serum was obtained from venous blood safely drawn from each participant and tested for specific anti-*Toxoplasma* antibodies IgG and IgM by ELISA. A serological criterion for seropositivity was a positive test result for any of the two anti-*Toxoplasma* antibodies or a combination of both. Questionnaire interviews were conducted to obtain personal information and *Toxoplasma* infection risk-related data.

**Results:**

Those who tested seropositive for anti-*T. gondii* antibodies were 51.2 % (64/125) pregnant women, 58.0 % (116/200) children and 57.6 % (72/125) HIV patients. The major risk factors associated with anti-*T. gondii* seropositivity were identified as age (in children), handling raw meat and gravida status (in pregnant women). The results of this study confirmed that the seroprevalence of *T. gondii* infection is high among pregnant women, hospitalized children <5 years old and HIV patients.

**Conclusions:**

A further study to investigate pre-pregnancy infections with *T. gondii* among women of childbearing age, seroconversion rate in pregnant women, rate of mother-to-child transmission and reactivated infections among HIV-seropositive persons is recommended.

## Background

Toxoplasmosis is one of the world’s most common parasitic infections caused by *Toxoplasma gondii*, a ubiquitous intracellular protozoan parasite of warm-blooded animals [1–3]. The parasite is known to infect most genera of warm-blooded animals, including humans, but the definitive host is the felids (cat). Toxoplasmosis is a self-limiting disease with the parasite encysting after few weeks of infection, but reactivation of infection may occur even in healthy individuals due to several factors including suppression of one’s immune system leading to significant morbidity and mortality [4, 5].

Seroepidemiological surveys have revealed varying degree of prevalence in terms of different geographical settings and the risk factors for acquiring the disease [6]. There have also been reports of varying prevalence in terms of age distribution, climate and socio-economic status [7–9]. Persons at risk of acquiring toxoplasmosis as an opportunistic disease usually are immune-compromised or immune-suppressed persons [10] due to the ability of the parasite to be reactivated from encysted bradyzoites to circulating tachyzoites [11].

In most European countries such as France and Austria, there are instituted national programmes that aim to screen pregnant women for toxoplasmosis, and such interventions have helped reduce the rate of congenital transmission and the rate of seroconversion due to early diagnosis [12, 13]. During pregnancy, there is the occurrence of a high cascade of complex physiological conditions that enables the mother to fully adapt to the environment. Immune-suppression is noted to be a physiological change that the body of the mother induces for the prevention of foetal rejection as the foetus is considered as an allograft. This, therefore, makes the pregnant woman susceptible to many opportunistic infections [14]. However, in Ghana, like most sub-Saharan African countries, there is no national programme for screening pregnant women for toxoplasmosis.

HIV infections on the other hand cause progressive depletion of the CD4^+^ T cells, which leads to life-threatening opportunistic infections (OIs) or malignancies during the natural course of the disease [15]. Thus, in chronic toxoplasmosis infected patients, there can be reactivation of a latent infection which can cause life-threatening encephalitis [16].

Primary infection of *T. gondii* during pregnancy can result in the vertical transmission of tachyzoites which can have severe consequences on the foetus such as retinochoroiditis, hydrocephalus and intracranial calcification of the children and long-term ocular lesions [17].

In Ghana, however, until recently when attention is being given to toxoplasmosis [18, 19], there is not much evidence-based information on *T. gondii* infections among high-risk groups in the populace. This therefore makes it difficult for the formulation and adoption of a national policy for toxoplasmosis management. We therefore sought to determine the level of exposure of infection among HIV-seropositive people, pregnant women and hospitalized children to *T. gondii* due to their suppressed or underdeveloped immune status.

## Methods

### Study area

The ante-natal unit, Obstetrics and Gynaecology Department and the Fevers Unit, Medical Department both at the Korle-Bu Teaching Hospital (KBTH) served as the recruiting sites for pregnant women and HIV-seropositive patients, respectively. KBTH is the leading tertiary referral hospital in Ghana with over 2000 bed capacity. The Princess Marie Louise Children’s Hospital (PMLCH), an exclusively referral children’s hospital in Accra, served as the site for recruiting children <5 years old.

### Study population

The study population consisted of pregnant women aged >18 years (in any trimester of pregnancy) attending ante-natal clinic, male and female Outpatient Department attendees of the Fevers Unit, aged above 18 years who were HIV-seropositive and not yet started treatment with antiretroviral therapy and all at the KBTH as well as children of both sexes aged <5 years admitted at the PMLCH.

### Sampling and sample size

For calculation of the sample size, we assumed a minimum of 10 % and a maximum 90 % *T. gondii* seroprevalence as expected frequency of the factor under study and a confidence level of 95 %. The result of the calculation was a minimum of 138 subjects for accurate statistical inference [20]. A convenience sampling method was used to sample for the participants, and elements of bias were controlled by ensuring that the sample was a miniature of the whole population under study.

### Sample collection

For participants aged above 18 years, 3–5 ml of blood was drawn from the cubital vein using a sterile hypodermic syringe, and the blood was transferred into labeled serum separator tubes. For children <5 years old, 3 ml of blood was obtained from heel prick and transferred into labeled serum separator tubes. All blood samples were transferred in cool boxes containing ice-packs to the laboratories of the Department of Parasitology, Noguchi Memorial Institute for Medical Research (NMIMR), for further analysis. Serum was obtained from each blood sample by centrifugation at 14,000 rpm for 10 min and stored at −20 °C until assayed for anti-*T. gondii* antibodies.

### Socio-demographic and toxoplasmosis risk-related data collection

Close-ended questionnaires were administered to collect demographic as well as toxoplasmosis-related risk factors data. Questionnaires were administered in an interview format in participants’ mother language for easy understanding.

Questions sought personal information on participants including their knowledge about the disease and exposure to possible *Toxoplasma* infection transmission risk factors. The possible infection transmission risk factors considered in the questionnaire included handling and eating of meat from high transmission risk animals (pigs, sheep and goats) and contact with cat. With reference to meat, they were asked how often they handled and/or ate the meat and what form they preferred their meat (thoroughly and/or partially cooked). The pregnant women and the parents of the children were also asked if they owned cats or pets or had cats in their house or immediate surroundings.

### Anti-*T. gondii* IgG and IgM detection by enzyme-linked immunosorbent assay

Each sample was tested for the presence of anti- *T. gondii* antibodies, IgG and IgM, using commercial ELISA kits (CTK Biotech. Inc., San Diego, CA, USA) and following manufacturer instructions. Briefly, 100 μl (for IgG) and 50 μl (for IgM) of sample diluent was added to the test wells of the 96-well microtitre plates (pre-coated with *T. gondii* antigen), and 10 μl of each test serum was added to each test well. The wells were then incubated at 37 °C for 30 min after which they were completely and thoroughly washed with wash buffer. One hundred microlitres of HRP-anti-human IgG conjugates or HRP-anti-human IgM conjugates (depending on the test) were added to each of the wells except the blank wells and incubated at 37 °C for 20 min after which wells were thoroughly washed. Fifty microlitres each of TMB (3, 3′, 5, 5′-tetramethylbenzidine) substrates A and B was added to the test wells. The 96-well microplate was then incubated in the dark for 10 min at 37 °C, and the reaction was stopped by adding 50 μl of stop buffer. Optical densities (OD) were then measured using a 450-nm ELISA plate reader, Multiskan™ Microplate Absorbance Plate Reader (Thermo Scientific, USA). The cut-off value was given as *N* + 0.15, where *N* was the mean OD of the negative control. The OD of each specimen was calculated as the ratio of the specimen OD and the cut-off value. The mean OD value of the *T gondii* IgG and IgM positive controls was expected to be ≥1.0 and that of negative controls was expected to be ≤0.10. Samples with an OD ratio <1.00 was considered to be negative with samples having an OD ratio ≥1.00 considered positive.

### Statistical analysis

Data analysis was performed using SPSS version 16.0. Difference in non-scalable variables such as age and sex of the study populations were assessed by Mann-Whitney *U* rank sum with *p* values <0.05 considered significant (CI 95 %). Chi-square (*χ*^2^) test was used to determine associations between categorical variables. *p* values <0.05 was considered statistically significant. Univariate analysis was used to predict the association between *T. gondii* seropositivity and risk factors.

## Results

### Participant characteristics

A total of four hundred and fifty (450) patients participated in this study. They comprised of 125 pregnant women aged 16 to 44 years (mean 28.25 ± 3.65) across trimesters; 200 children aged 0 to 5 years (mean 2.62 ± 1.31); and 125 HIV-seropositive persons aged 15 to 68 years (mean 43.24 ± 4.97) (Table 1). There was a significant difference between the ages of the study groups.

**Table 1:** General characteristics of study participants

		Study participants		
		Pregnant women	Children <5 years old	HIV-seropositive
	Number sampled	125	200	125
Age (years)	Range	16–44	0–5	15–68
	Mean	28.25 ± 3.65	2.62 ± 1.31	43.24 ± 4.97
Gender	Male (%)	–	109 (54.5)	54 (43.2)
	Female (%)	125 (100)	91 (45.5)	71 (56.8)

### Prevalence of anti-*T. gondii* IgG and IgM antibodies

Seroprevalence of anti -*T. gondii* IgG among pregnant women, children and HIV-seropositive persons were 51.2 % (64/125), 58.0 % (116/200) and 57.6 % (72/125), respectively. A seroprevalence of 0.5 % (1/200) anti-*T. gondii* IgM was recorded among the children. However, there was no record of anti-*T. gondii* IgM in pregnant women and HIV-seropositive persons (Table 2).

**Table 2:** Seroprevalence of anti-*Toxoplasma gondii* antibodies among study participants

*T. gondii* seropositivity *n* (%)			
Study participants	Number tested	IgG only	Both IgG and IgM
Pregnant women	125	64 (51.2)	0
Children	200	116 (58.0)	1 (0.5)
HIV-seropositive	125	72 (57.6)	0

### Test of association between anti-*T. gondii* seropositivity and socio-demographic variables in pregnant women and HIV-seropositive persons

Demographic and clinical factors were related to seropositivity of anti-*T. gondii* antibodies. Results indicated no significant association between gender and the seropositivity for anti-*T. gondii* IgG. Furthermore, factors such as age (all study participants), stage of pregnancy and CD4^+^ T cell count/mm^3^ were found not to have any statistical significance among pregnant women and HIV-seropositive persons, respectively. However, there was a significant association between the gravida status of the pregnant women and anti-*T. gondii* seropositivity (Table 3). Exposure to infection risk factors such as owning a cat and contact with soil (due to their daily activities) as well as the handling and eating of raw or undercooked meat did not show any statistically significant associations with anti-*T. gondii* seropositivity in both pregnant women and HIV-seropositive persons. However, there was a significant association between handling of meat (beef, pork, mutton and chevron; among pregnant women only) and anti-*T. gondii* IgG positivity (*p* = 0.02; CI 95 %) (Table 4).

**Table 3:** Demographics associated with *Toxoplasma* seropositivity in participants by univariate analysis

Study participants	Number examined (*N*)	Anti-*Toxoplasma gondii* seropositivity (*n*/*N*; %)	*χ* ^2^ value	*p* value (CI 95 %)
Pregnant women	125	64 (51.2)		
Age groups				
16–20	4	4 (100)	3.94	<0.05*
21–30	57	30 (52.6)	0.08	0.76
31–40	58	27 (46.6)	0.94	0.33
41–44	6	3 (50.0)	0.04	0.95
Stage of pregnancy				
1st trimester	7	4 (57.1)	0.11	0.74
2nd trimester	38	22 (57.8)	0.98	0.32
3rd trimester	80	38 (47.5)	1.21	0.26
Gravida status of women				
Primigravids	49	17 (34.7)	8.79	0.003*
Multigravids	76	47 (61.8)		
HIV-seropositive persons	125	72 (57.6)		
Age groups				
15–24	4	1 (25.0)	1.79	0.17
25–34	27	16 (59.3)	0.04	0.84
35–44	51	26 (50.9)	1.54	0.21
45–54	30	20 (66.7)	1.32	0.25
55–64	12	8 (66.7)	0.44	0.50
65–68	1	1 (100)	0.74	0.389
Gender				
Male	54	32 (59.2)	0.10	0.74
Female	71	40 (56.3)		
CD4^+^ T cell count cell/mm^3^				
<200	9	6 (66.7)	0.32	0.56
200 ≥ CD4^+^ ≤ 500	64	38 (59.4)	0.16	0.68
>500	52	28 (53.8)	0.52	0.47
Children <5 years of age	200	117 (58.5)		
Age groups				
0–6 months	27	6 (22.2)	26.46	<0.001*
6–12 months	30	9 (30.0)	11.80	<0.001*
1–3 years	69	48 (69.6)	5.31	0.02*
3 years > X < 5 years	74	53 (71.6)	7.32	<0.001*
Gender				
Male	109	68 (62.4)	1.18	0.27
Female	91	49 (53.8)		

*p* value was calculated by Pearson’s chi-square (*χ* ^2^) with confidence interval (CI) of 95 % and 1 degree of freedom (*df*). *p* values less than 0.05 were considered statistically significant and are indicated in bold fonts (*)

**Table 4:** Association of infection risk variables with *Toxoplasma gondii* seropositivity in pregnant women and HIV patients by univariate analysis

Study participants	Factor	Number examined, *N* (*N*/*N*_0_; %)	Anti-*Toxoplasma gondii* seropositivity, *n* (*n*/*N*; %)	*χ* ^2^ value	*p* value (CI 95 %)
Pregnant women (*N*_0_ = 125)	Own a cat				
	Yes	56 (44.8)	28 (50.0)	0.058	0.808
	No	69 (55.2)	36 (52.1)		
	Contact with soil				
	Yes	14 (11.2)	7 (50.0)	0.009	0.92
	No	111 (88.8)	57 (51.4)		
	Handle raw meat				
	Yes	114 (91.2)	62 (54.4)	5.26	0.02*
	No	11 (8.8)	2 (18.2)		
	Eat raw meat				
	Yes	28 (22.4)	14 (50.0)	0.02	0.88
	No	97 (77.6)	50 (51.5)		
HIV-seropositive patients (*N*_0_ = 125)	Own a cat				
	Yes	42 (33.6)	26 (62.0)	0.47	0.48
	No	83 (64.4)	46 (55.4)		
	Contact with soil				
	Yes	64 (51.2)	35 (54.7)	0.45	0.49
	No	61 (48.8)	37 (60.7)		
	Handle raw meat				
	Yes	75 (60.0)	40 (53.3)	1.39	0.23
	No	50 (40.0)	32 (64.0)		
	Eat raw meat				
	Yes	111 (88.8)	66 (59.5)	1.40	0.23
	No	14 (11.2)	6 (42.9)		

*p* value was calculated by Pearson’s chi-square (*χ* ^2^) with confidence interval (CI) of 95 % and 1 degree of freedom (*df*). *p* values less than 0.05 were considered statistically significant and are indicated in bold fonts (*)

### Prevalence of anti-*T. gondii* antibodies in children

Children in all age groups tested for anti-*T. gondii* antibodies showed a significant association with age and seropositivity with *p* values <0.05 (CI 95 %). Results indicated an apparent linear increase in seropositives in the different ages of the children as shown in the Table 3. The lowest seroprevalence of 22.2 % (6/27) was recorded in the 0- to 0.5-year-old group and is likely to be circulating maternal antibodies and steadily increased in the other age groups with the highest seroprevalence of 71.6 % (53/74) being recorded in children aged between 3 and 5 years.

## Discussion

The current study was conducted to determine the seroprevalence of anti-*T. gondii* antibodies among immune-suppressed groups of people represented by pregnant women, children and HIV-seropositive persons in Accra. All three groups recorded serum anti-*T. gondii* antibodies positivity of more than 50 %. A previous study among pregnant women (mean age of 28.1 years) in Accra, Ghana, yielded a seroprevalence of 92.5 % [18]. However, another hospital-based study in patients of mean age 30.2 years visiting the Korle-Bu Teaching Hospital in Accra reported a lower prevalence rate of 49.7 % [19]. A recent population-based study in coastal Ghana also reported a seroprevalence of 85 % [8]. These disparities in toxoplasmosis seroprevalence estimates could be due to several factors such as study and geographical and climatic factors which are known to influence seroepidemiological studies [21]. Similar studies in other African countries have revealed varying prevalence such as 58.4 % in Tunisia [22], 53.6 % in Benin [23] and 57.9 % in Egypt [24].

This study showed no significant association between anti-*T. gondii* antibody detection and presence of domestic cats, and this is in contrast with the results of studies reported in Nigeria [25], India [26] and Tanzania [27]. As IgM is considered as an indicator for recent/acute infection, our data suggests that there were no recent active infections in the pregnant women nor HIV-seropositive persons. The non-detection of IgM suggests that there were no recent infections; however, the presence of IgG could be due to reactivated or past infections [28].

It is crucial in pre- natal screening to ascertain whether *T. gondii* infection was acquired before or after conception [12, 14]. Primary congenital toxoplasmosis can lead to parasite transmission to the foetus via the placenta where the risk of transmission increases during gestation [29]. In seroepidemiological studies for *T. gondii* infection, the detection of anti-IgM antibodies in combination with anti-IgG antibodies is always an indication of acute infections [30]. The reason is that IgM antibodies are not usually in acquired immunity and very rare in chronic infections. Furthermore, the presence of IgG together with IgM in circulation indicates recent acquired infection because; IgM antibodies wane down rapidly following recently acquired infections [18].

The current study recorded an overall seroprevalence of anti-*T. gondii* infection in children to be 58.5 % for anti-*T. gondii* IgM and/or anti-*T. gondii* IgG. One (1) male infant aged 4 years tested positive for anti-*T. gondii* IgM. This is an indication of primary infection. Considering his age infection, he must have acquired the infection through exposure to any of the potential related risk factors. The seroprevalence recorded in this study was higher than the values reported for children of similar ages in a Nigerian study which reported 23.8 % seroprevalence [31]. The difference in seropositivity rates could probably be due to the diagnostic techniques used which has variable sensitivities and specificities and also due to the local potential relative risk factors associated with the geographical areas.

This study found a statistically significant association between seroprevalence and age in children. Findings from the study showed a linear increase from the <6 months old children which recorded a 22.2 % seroprevalence to the 3- to 5-year-old children which recorded a seroprevalence of 71.6 %. This observation was in agreement with studies done elsewhere, in which the seroprevalence of *T. gondii* infection was found to increase with age [10, 32]. Previous studies had observed that there was a rapid increase in anti-*T. gondii* IgG seroprevalence from ages 6 months to 5 years and then plateaued through age 10 years [33]. One hypothesis is that this trend simply reflects the increasing number of “exposure years” as children aged [33]. As children tend to grow, they get in contact with soil which is known to be a high-risk factor in terms of exposure to *T. gondii* oocysts. Also in children <6-month-old age group, the IgG antibodies found are likely to be circulating maternal antibodies which has been passively passed on to the children or may be due to congenital infection. Establishment of congenital transmission however depends on the detection of parasite DNA in the foeto-placental region of the placenta [1, 28, 34], and thus, anti-*T. gondii* antibodies will not be a conclusive diagnostic test.

Although pre-pregnancy *T. gondii* infection status of the pregnant women is unknown, it could be deduced from the antibodies detected and age of pregnancy that there is a possibility of having acquired the infection before conception or during pregnancy. Previous studies have shown that *T. gondii* infections acquired by the mother during the first trimester of gestation has a 10–15 % risk of being transmitted congenitally to the foetus with severe consequences [14] whereas mothers contracting the infection during the second or third trimesters has an increasing (up to 68 %) risk of infecting their unborn babies, with less severe consequences [14]. This is therefore an indication that almost all the seropositive pregnant women identified in this study stand a risk of transmitting the infection to their foetuses. However, this study cannot conclusively ascertain whether there were active infections ongoing in the pregnant women and as such the rate of congenital transmission cannot be determined.

Toxoplasmosis is the most common opportunistic infection in HIV-seropositive immune-compromised hosts, where it occurs predominantly as a reactivation of endogenous infection [35].

Until the last couple of decades, most human *T. gondii* infections were thought to be the result of contact with soil contaminated with the oocysts, since *T. gondii* oocysts can survive for years in soil [4]. Recently, many infections are thought to result from other related risk factors including cat ownership, eating raw or undercooked pork, meat and meat products, consumption of raw and unwashed vegetables [8, 9] and drinking from contaminated reservoir [36].

In the current study, some variables were identified as possible infection risk factors and were independently linked to a higher risk of *T. gondii* infection. The handling of raw meat (which includes beef and pork) among pregnant women had statistically significant association with anti-*T. gondii* seropositivity as a higher proportion of the pregnant women that reported handling raw meat (62, 54.4 %; *N* = 114) had anti-*T. gondii* antibodies than the non-raw meat handlers (2, 18.2 %; *N* = 11). Handling of raw meat was however not a significant risk factor in HIV-seropositive patients as the proportion of non-raw meat handlers with anti-*T. gondii* seropositivity (32, 64.0 %; *N* = 50) was higher than the raw meat handlers (40, 53.3 %; *N* = 75) although the number of the latter was higher than the former (Table 4). *Toxoplasma* tissue cysts contained in meat or meat-derived products have been shown to serve as important sources of infection for humans [8] and that the risk of acquiring the infection via meat sources depends on cultural and eating habits in different human populations. Cat ownership did not show any significant association with *T. gondii* seropositivity in this study. These findings are in contrast to those of previous studies [8, 37] which observed that cats play a central role in the epidemiology of *T. gondii*, constituting the only known source of environmental contamination with the infective oocyst stage. However, in support of our findings, other epidemiological studies have not shown cat ownership to be a high-risk factor for *T. gondii* infection [38] but rather exposure to the faeces from a cat that is shedding oocysts.

The seemingly high seroprevalence recorded in the children as compared to the two adult study populations may be possibly due to a variety of factors. Firstly, it is possible that the children had been exposed to risk factors that adult populations in this study had not been exposed to during their childhood. With the increase in urbanization and industrialization in many parts of Ghana, it is possible that there is an increased risk for *T. gondii* infection. The increase in urban slums is a potential high-risk point for disease transmission including *T. gondii* because of inaccessibility to clean water, unhygienic practices and inadequate sanitation. Also, the recruitment at an exclusively referral children’s hospital may be a confounding factor and that results obtained may not generally be same for supposedly healthy children in the country.

## Conclusions

In conclusion, the results of this study confirmed that the seroprevalence of *T. gondii* infection is high among pregnant women, children <5 years old and HIV-seropositive persons in Accra. The major risk factors associated with *T. gondii* seropositivity in the present study were age and contact with raw meat (among only pregnant women). A further study to investigate pre-pregnancy infections with *T. gondii* among women of childbearing age, seroconversion rate in pregnant women, rate of mother-to-child transmission and reactivated infections among HIV-seropositive persons is recommended. Such evidence will add up to existing information to improve control and management of toxoplasmosis in Ghana, especially for women of childbearing age in Ghana.
